# Algorithmic fairness: challenges to building an effective regulatory regime

**DOI:** 10.3389/frai.2025.1637134

**Published:** 2025-08-29

**Authors:** Greg Demirchyan

**Affiliations:** Fairlogic.ai, Pasadena, CA, United States

**Keywords:** artificial intelligence, algorithmic fairness, fairness definitions, proposed legislation, bias, discrimination, protected groups, adverse impact

## Abstract

Unfair treatment by artificial intelligence toward protected groups has become an important topic of discussion. Its potential for causing harm has spurred many to think that legislation aimed at regulating AI systems is essential. In the US, laws have already been proposed both by Congress as well as by several key states. However, a number of challenges stand in the way of effective legislation. Proposed laws mandating testing for fairness must articulate clear positions on how fairness is defined. But the task of selecting a suitable definition (or definitions) of fairness is not a simple one. Experts in AI continue to disagree as to what constitutes algorithmic fairness, which has led to an ever-expanding list of definitions that are highly technical in nature and require expertise that most legislators simply do not possess. Complicating things further, several of the proposed definitions are incommensurable with one another, making a cross-jurisdictional regulatory regime incorporating different standards of fairness susceptible to inconsistent determinations. On top of all this, legislators must also contend with existing laws prohibiting group-based discrimination that codify conceptions of fairness that may not be suitable for evaluating certain algorithms. In this article, I examine these challenges in detail, and suggest ways to deal with them such that the regulatory regime that emerges is one that is more effective in carrying out its intended purpose.

## 1 Introduction

Fairness is an important moral dimension we use to evaluate the impact that society has on our lives. And so it is not surprising that we desire to see fairness extend to artificial intelligence. Unfair treatment by artificial intelligence has garnered significant attention, especially when directed at historically vulnerable groups in the form of bias or discrimination. Wary of these dangers, many want to see legislation passed, and urge in particular the assessment of AI-generated automated systems for fairness. In the US, both Congress as well as a number of states have already proposed laws that would require those responsible for developing or deploying certain AI systems^1^ to also assess them for fairness, and, if necessary, to take remedial measures and rid their algorithms of these harms.^2^

Of course, when proposing laws requiring fairness in AI systems and their algorithms, it is important that these be consistently and predictably applied. If laws are to require testing for fairness, we must also have a suitable definition or definitions of fairness. Where a particular definition has a specific area of application and its satisfaction in this context would determine fairness, we’ll likely have to settle for multiple definitions or standards. Thus, it becomes incumbent on the legislators to provide the correct definition, and—if multiple definitions are involved—the appropriate scope must be defined for each.

So where would we look for definitions of fairness? Fairness is a complex notion subject to competing interpretations, deeply personal to us, and our sense of it is as much a reflection of our individual outlook as the society we live in. Appealing to the general public is unlikely to yield an authoritative answer, because though we have strong beliefs and do not hesitate to make them known, most of us would be hard-pressed to provide a conception of fairness that organizes our intuitions about this subject—and even if we could, it likely would not reflect anything like a consensus view. As members of a pluralist society, ascribing to a variety of political values, our views about fairness do not coalesce into anything like a stable conception.

What about the experts? AI experts come from fields ranging from computer science to sociology and law. As their efforts to define algorithmic fairness are influenced both by training and background, the picture that emerges is anything but clear.^3^ Despite robust discussion, a number of conceptual difficulties make convergence unlikely. On the contrary, differing opinions often lead to the introduction of additional definitions, further complicating matters for legislators looking for clear answers. So reliance on experts will also not yield tidy and uncontroversial answers on fairness.

My objective here is to explore some of the challenges to devising a coherent and internally consistent regulatory regime, as legislators unaware of them may propose legislation that inadvertently smuggles in various conceptual problems, making regulating for fairness a task fraught with ambiguity and uncertainty. I contend that legislators must confront and articulate clear positions on the issues presented by these challenges if they are to develop a stable, transparent, and effective regulatory regime.

## 2 Proposed AI laws

Recently, a spate of proposed AI legislation has been introduced in Congress and several state legislatures. A number of these proposed bills show specific concern regarding how protected groups may be treated by artificial intelligence and include provisions meant to prevent such groups from being unfairly disadvantaged.^4^ For instance, the proposed laws uniformly mandate initial and periodic impact assessments of certain automated decision systems which must include among other things evaluating these systems for any foreseeable risk of algorithmic discrimination. And they forbid the deployment of automated decision systems that have been assessed to result in algorithmic discrimination in the form of “unjustified” differential treatment or impact.^5^

What stands out about these proposed laws is that despite decrying group differences and making algorithmic fairness a requirement, not one offers a clear statement on how algorithmic fairness is being defined––nor do these laws directly refer to any of the numerous definitions of fairness that have been developed by experts working on this subject.^6^ But the absence of a clearly articulated position on fairness should be a cause for concern. In particular, laws that sidestep this issue run the risk of creating a regulatory regime that will face a number of problems, as I will suggest.

### 2.1 Ubiquity of adverse impact

A common thread running through these bills is the significance they give to adverse impact. A number of important U. S. civil rights laws that oppose discrimination in employment, housing, and other areas view adverse impact^7^ as posing a serious risk of unfair treatment.^8^ Seemingly neutral policies or practices may nevertheless have consequences where protected groups are negatively and disproportionately affected. It is also important to note that these civil rights laws emerged at a time when artificial intelligence was still a fledgling field with little relevance outside academia. And adverse impact ended up being about finding group differences in selection rates emanating from selection decisions made by people. Things have become more complicated with the advent of predictive algorithms derived from machine learning. Thanks to powerful statistical methods, we have many more parameters with respect to which group differences can be measured. We may find group differences in selection rates. But there may also be group differences in false positive rates or in the accuracy of an algorithm’s predictions. Every one of these differences can plausibly be considered an example of adverse impact. What’s more, there is no way that a predictive algorithm can equalize results across all these parameters. Moreover, equalizing results with respect to some particular dimension often responds to the demands of a particular definition of fairness. With so many definitions of fairness and so many ways to manifest group differences, any algorithm may be found to be complicit in generating adverse impact of some form.

The current language of the laws does not prohibit one from simply choosing some definition (or definitions) of fairness. This of course can open the door to gaming the system. Developers interested in protecting their algorithms from being accused of bias can cherry pick a fairness definition that shows their algorithm is perfectly fine. Those who are intent on showing a certain algorithm is discriminatory can find a definition the algorithm fails to satisfy. Without some kind of a filter as to which definitions should be allowed, every case where an algorithm’s fairness is being assessed from a legal standpoint becomes a matter of debate, which is poor way to deal with any legal issue, especially something of this magnitude.

An additional problem is the elision in the statutes of any kind of procedure that would provide a way to overcome a prima facie case of group discrimination where there is adverse impact. Without a way to overcome a claim of discrimination in some cases, in every situation where there is adverse impact––which is hard to eschew given how ubiquitous adverse impact can be when there is no gatekeeping on what counts as algorithmic fairness––it becomes hard to avoid the conclusion of unlawful discrimination.

It may be argued that these laws, though they are not explicit about it, nevertheless intend to construe adverse impact in pretty much the same way that civil rights laws do. Under Title VII, adverse impact is measured in terms of group differences in selection rates of employment-related selection procedures (Title VII of the Civil Rights Act, 1964). And so, the argument would go, these proposed laws are intended to follow suit and define adverse impact as group differences in selection rates. What is interesting about assessing selection decisions in terms of their selection rates across groups is that this is an application of statistical parity, a definition of fairness that has received a good deal of critical attention. According to statistical parity, fairness is achieved if the marginal distribution of predicted classes––i.e., job applicants who are hired by an employer, inmates who are predicted not to recidivate if released on parole, college applicants who are predicted to succeed in college––is the same for all protected groups. Adverse impact in the form of group differences in selection rates violates the fairness requirement of statistical parity.

There are a few things that can be said in response to such an argument. The crucible of statistical analysis has given us an array of possible ways in which adverse impact may be interpreted. With such a variety of ways to construe adverse impact, the mere mention of it does not point to any particular definition. So, if the proposed laws intend for adverse impact to be interpreted in more or less the same manner as traditional civil rights laws do, then they should state this explicitly. Furthermore, if statistical parity is meant to be the operative definition of fairness, one should tread with some caution. Statistical parity has been criticized for presenting a rather simplistic view of fairness. When applied in more complex situations, statistical parity at times may give a counterintuitive result, at others it may seem entirely misplaced. Let us look at each of these possibilities separately.

Not all group differences in selection rates should count as unlawful discrimination––sometimes group differences in predicted outcomes can be based on legitimate grounds. It is a well-known fact, reflected in official crime statistics, that there is a gender gap in the commission of violent crimes: men commit more violent crimes than women; men are also more likely to reoffend (CDE, 2025). A number of theories have been offered to explain the gender gap in violent crime, but none point to discrimination as a causal factor (Cullen and Lero Jonson, 2011; Daly, 1994). If presented with an algorithm that predicts different rates of recidivism between men and women, we would not think there is anything unfair or discriminatory about these results since the underlying cause for this difference is not linked to discriminatory treatment of men. But statistical parity would say otherwise, for it does not tolerate differences as non-discriminatory.

As mentioned before, civil rights laws require a more extensive procedural analysis before adverse impact is cognized as an unlawful form of group discrimination (see Romei and Ruggieri, 2014). Under Title VII, where a certain employment practice results in adverse impact, a claim of unlawful discrimination may nonetheless be overcome if the employer is able to show that the applied practice is necessary for evaluating job-related qualifications and there are no alternatives that have less of an adverse impact (Title VII of the Civil Rights Act, 1964; Equal Employment Opportunity Commission (EEOC), Civil Service Commission, Department of Labor, and Department of Justice, 1978). Similarly, claims of disparate impact are cognizable under the Fair Housing Act. Policies that result in a disproportionate adverse effect on a protected group are deemed unlawfully discriminatory unless they can be justified by legitimate and nondiscriminatory reasons (Fair Housing Act, 1968). Adverse impact as a violation of statistical parity only establishes a prima facie case of discrimination, which may be overcome if there are sufficiently compelling justifying reasons. The proposed laws on AI do not include any provisions for further analysis.^9^ And strict reliance on statistical parity will result in many algorithms being deemed unlawfully discriminatory when common sense would say otherwise. In fact, common sense would say that when it comes to recidivism rates between men and women, engineering a predictive algorithm so that it satisfies statistical parity would actually result in unfairness to women. In many situations, statistical parity can be a poor choice for determining fairness.

Another problem with statistical parity is that it can be used to assess only certain types of algorithms and going beyond its limited scope of application may culminate in harm to members of vulnerable groups. Several of the proposed bills cover algorithms that make decisions affecting a person’s access to healthcare, education, criminal justice, and financial services.^10^ For some of these algorithms, statistical parity is categorically the wrong approach to take for assessing fairness. Assume we are evaluating an algorithm that tries to predict whether an individual exhibiting certain symptoms has a life-threatening condition. As it happens, many more members of group A are predicted to have this condition than members of group B. Here we do not have parity in the prediction rates. But it would be wrong to conclude that ipso facto there is something unfair about these results: it may well be the case that members of A for whatever reason are more vulnerable to succumbing to this life-threatening condition. If the algorithm is doing a good job of predicting the presence of this condition, equalizing the prediction rates between the two groups would not achieve a fairer result. In fact, it would likely cause less accurate predictions and thus endanger the lives of the more vulnerable members. We still think that fairness is relevant in this case. But in a diagnostic setting, accuracy matters a great deal and serves as a critical reference point for conceptualizing fair treatment. When assessing an algorithm that has a diagnostic function, statistical parity is simply not the correct standard to apply.

Legislators must be aware that certain definitions have their limitations, and if they are to incorporate them into laws, they must make sure these definitions are used on algorithms they are suited to assess. The proposed laws do not specify these limitations. If they are intending to invoke statistical parity as their operative definition for assessing algorithms that are used as diagnostic tools, forcing equality in prediction rates on these algorithms will only end up causing harm. To avoid this requires giving up on statistical parity or designing legislation that establishes a clear division of labor among definitions, with each definition being matched to a set of algorithms that can be properly evaluated by it.

### 2.2 Fairness definitions

Over the last decade, judges and parole boards have become increasingly reliant on model predictions of recidivism in the form of risk scores when making decisions regarding the length of a sentence to be imposed or whether to grant a defendant an early release from prison. Defendants who receive risk scores that determined them to be at a high risk for recidivism are denied a chance to have a lighter sentence or an early release. Many risk assessment tools have been developed for such a task and one of the more prominent has been COMPAS, which has also drawn the most attention from critics.

In 2016, ProPublica published a report which examined risk scores assigned by COMPAS to over 10,000 individuals arrested in Broward County, Florida (Angwin et al., 2016).^11^ The authors found that “Black defendants who did not recidivate over a two-year period were nearly twice as likely to be misclassified as higher risk compared to their white counterparts” (Angwin et al., 2016). Moreover, White defendants who re-offended over the same time period were misclassified as low-risk almost twice as often as Black recidivists. As a result, more White recidivists received a lighter sentence or were granted parole compared to their Black counterparts. In other words, ProPublica’s analysis found that COMPAS generated more false positives and fewer false negatives for Black defendants but fewer false positives and more false negatives for White defendants. ProPublica concluded that this disparity meant COMPAS is deeply unfair to African Americans.

In its criticisms of COMPAS, ProPublica adopted a conception of algorithmic fairness that at the very least requires parity in false positive and false negative rates––or error rates––between Black and White defendants. Several influential definitions of algorithmic fairness have been developed focusing on the error-rate aspect of a predictive algorithm’s output. One such definition––“equalized odds”––calls for equalizing both false positive as well as false negative rates across groups and perhaps comes closest to ProPublica’s understanding of fairness.^12^ Other definitions of similar ilk hone in on equalizing either the false positive or the false negative rates (Berk et al., 2017).

In response, COMPAS’s maker, Northpointe, argued that evaluating the algorithm from the perspective of error rates is not the right way to assess bias (Dieterich et al., 2016). Instead, an algorithm is fair if it achieves parity in its positive predictive value across all relevant protected groups (Dieterich et al., 2016).^13^ Northpointe and others were able to show that COMPAS is free of racial bias under a conception of fairness that prioritizes equality of positive predictive value (Dieterich et al., 2016). A number of important definitions of fairness have sprung around the idea of comparing the accuracy of predicted outcomes for all relevant groups. Predictive parity, which is satisfied if the fraction of correct positive predictions (e.g., will recidivate) is the same across all relevant groups, aligns most closely with Northpointe’s approach to fairness.^14^

ProPublica and Northpointe clearly construe fairness in different ways. But we can still ask the following question: why cannot an algorithm exhibit parity along both dimensions—positive predictive value *and* error rates? If COMPAS could simultaneously satisfy both standards, it would certainly make things simpler. The answer unfortunately is that satisfying these definitions simultaneously is not logically possible. Under conditions that prevail in the US, some definitions of fairness are simply incommensurable with one another. Group differences in prevalence rates of recidivism create an imbalance between positive predictive value and error rates, such that equalizing them across groups is not possible.^15^ The set of Black and White defendants from Broward County exhibit different prevalence rates of recidivism, with Black defendants having a higher rate. Where group differences in prevalence rates prevail (which is the case for most real-world scenarios), we face a choice. We can require the algorithm to instantiate equality of positive predictive value or equality of error rates across groups but we cannot have both. If we constrain COMPAS to satisfy positive predictive value parity for Black and White defendants, the algorithm cannot also equalize the error rates.^16^

Incommensurability among definitions of fairness poses a profound challenge to lawmakers who wish to regulate AI systems for fairness. The debate between ProPublica and Northpointe illustrates that when it comes to regulating predictive algorithms for fairness, making hard choices is inevitable. Whichever choice is made there will be controversy, for our intuitions do seem to be pulled in different directions. ProPublica’s moral disdain over the disproportionate number of Black defendants incorrectly predicted to be recidivists packs a powerful punch: we think it is particularly odious to punish someone for something they did not do. Moral reasons can also be adduced for choosing equality of positive predictive value. There are clear moral costs to sacrificing equality of positive predictive value for the sake of achieving inter-group equality of error rates: prioritizing equality of error rates over equality of positive predictive value results in less accurate predictions for groups that have lower base rates. In the context of criminal justice, this can have a significant moral cost. Groups that end up on the losing end of unequal positive predictive value have compelling grounds to claim that they are not being treated fairly.

Despite the fact that no definition of fairness is capable of satisfying all of our intuitions about fair outcomes, it is nevertheless critical for lawmakers to pass laws that have a clear and coherent position on fairness, even if this means choosing a definition (or set of definitions) that will not receive universal acceptance. Having a regulatory regime encumbered by incommensurable definitions will make compliance with the requirements of algorithmic fairness, borne out of these definitions, simply unachievable. This applies not only within specific pieces of legislations but cross-jurisdictionally as well: states must ensure that when it comes to regulating AI, their laws and the laws of other states do not end up codifying standards of fairness that make simultaneous compliance with the laws of each state an impossible task. Achieving consistent and commensurable standards of algorithmic fairness cross-jurisdictionally will undoubtedly require some amount of coordination and cooperation among states interested in establishing their own regulatory system on AI. Of course, recognizing that incommensurability is an issue that must be contended with is the critical first step.^17^

## 3 AI and accountability

In addition to selecting a suitable fairness definition, there is the matter of identifying those who should be held responsible for setting things right when algorithms fail the test of fairness. Who is ultimately accountable depends on how fairness is defined. I contend that identifying the accountable parties is not always a straightforward matter. This might seem surprising. After all, when it comes to holding someone accountable for moral harms, is not the developer the obvious choice?^18^ Not always. In situations where the unfairness of an algorithm’s output depends on certain facts about our society, justifying the attribution of blame solely to the developer––and making them the agent responsible for mitigating this harm––has its challenges from the standpoint of fairness. I suggest that a more egalitarian and broader notion of accountability may be easier to justify and will also be more effective against unfair treatment in the long run. But before we examine the issue in more detail, a brief excursion into how algorithms can bring about an unfair result in contravention of a particular definition of fairness is in order. Let us consider equality of predictive value as our definition of fairness, and—in turn—how this definition can produce unfair results. Let us also assume that the algorithm that has been developed––and is being scrutinized here––prognosticates recidivism between Black and White defendants in the US.

First, showing that predictive values differ across groups does not yet tell us how and why these differences came about. In current practice, most algorithms that are products of machine learning are like black boxes; the processes that generate predictions are extremely opaque and it is still quite difficult even to experts to understand how algorithms generate their predictions. In fact, what counts as interpreting or deciphering such algorithms is a problem that remains unresolved. So while a detailed accounting is not yet possible, we can say that whatever caused the difference will be found within the four corners of the data that is been ingested and the emergent processes that define the algorithm. We do not have to bring in a distant exogenous factor to explain how the inequality originated. Because the source of the inequality of predictive values is internal to the development process, the developer’s decisions clearly matter: they control its design, development, and deployment.^19^ If rectifying the algorithm is technically feasible, it is fitting to hold the developer responsible for the unfair output, and for correcting it.

The point here is that when forms of algorithmic unfairness are linked to the algorithm’s origin and thus a product of the developer’s design and development choices, we have compelling grounds to assert that the algorithm’s developer should be held responsible for any harm that they cause, and that laws reflecting such accountability are justified. Because their acts or omissions play a critical role, and the unfairness is essentially endogenous to the process of creating the algorithm, these cases fall in line with our intuition that when algorithms cause harm, the buck stops principally with the developer.

Now let us compare our analysis of accountability to a contrasting situation—one in which fairness is defined by equality of error rates. Assume the algorithm violates this standard because it is well-calibrated and we live in a society where there are differences in recidivism rates.^20^ In this situation, unlike the previous one, there is an exogenous factor that is contributing to the algorithm’s failure to achieve equality of error rates between Black and White defendants. And that factor is the difference in prevalence rates between these groups. This difference is exogenous precisely because it is outside the sphere of the developer’s agency. The complication here is that the unfairness that results is not wholly of the algorithm’s making.

Making sense of the intuition that an inequality in error rates is unfair requires us to foreground the broader social context in which the algorithm operates, and relevant for conceptualizing this social context is that protected groups appear to exhibit different recidivism prevalence rates. Of course, we must do more than register the presence of this difference to explain why we might consider it unfair, and furthermore, we have not yet said anything about the moral status of this inequality. To motivate our moral outrage at the disparity, we have to understand the difference in prevalence rates as itself a product of injustice, a reflection of racial disparities shaped by the history of discrimination against Black Americans. Our turn toward moral condemnation is based on recognizing that Black Americans have suffered from a history of injustice that continues to leave its mark.

One might argue that imposing a legal duty solely on the developer to rectify a result that ultimately supervenes on a complex set of social conditions shaped by a history of racial oppression might seem—at least prima facie—somewhat one-sided and unfair. But then the question is, what would be fair terms of accountability under social conditions that have been affected by racial injustice? Formulating policies that aim to eliminate the conditions that cause them, through collective action on the part of the American polity, is bound to be more effective. In fact, this would be in line with how similar problems are handled outside the context of artificial intelligence. On a daily basis, human decision-makers face similar predicaments in other arenas. Although we have a duty not to commit discrimination, we have no positive duty to undo the effects of past injustices in every situation where we are presented with an opportunity to do so. This does not mean, however, that there is no positive duty as such at all, only that the obligation to address an injustice applies selectively and must be done in a way that expresses fair terms. As usual, the hard problem is coming up with these terms, and—in general—this process involves broad-based social participation. Of course, such terms can still adduce obligations that are directed specifically at developers. The important point is that eliminating disparities that contribute to this particular form of algorithmic unfairness should not absolve the American public of its responsibility. Shared and collective accountability would exemplify fairer terms of social cooperation and also has the further advantage of being capable of effectuating policies that address the deeper and more resistant sources of group disparities that still exist in our society.^21^

## 4 Algorithmic unfairness and the broader social context

As we saw in the previous section, predictive algorithms do not function in isolation. Instead, they operate within a complex set of systems involving forms of participation and cooperation on terms that have been established through extensive social and political processes, where the choices and views of the more privileged—on what makes something fair and just, for instance—typically dominate. The recognition that participation in our social and political institutions takes place on unequal terms, which then create and perpetuate conditions that result in certain groups being advantaged while others are disadvantaged, implies that there is broader social context within which algorithms are developed and their fairness is assessed. Invariably this broader context incorporates norms and decisions that can be morally questionable—because, for instance, they are based on harmful societal biases. More importantly, the power dynamics implicit in the broader context where predictive algorithms are applied are such that challenging problematic norms and biases is not something that can be achieved through compliance with statistical measures of fairness. Indeed, it is possible to comply with a statistical definition of fairness and still end up with an unfair outcome. Thinking that statistical measures are enough to achieve a fair outcome is to fall into what is called the formalism trap (Selbst et al., 2019). One must go beyond formal definitions of fairness and address the conditions and norms that govern such things as who decides what counts as a fair algorithm, and whether choices about model form and parameters are based on sound assertions. If there are groups who are directly affected by the results of predictive algorithms and yet remain sidelined and voiceless, this will have important ramifications for assessing fairness outcomes.

For instance, in the context of a criminal justice system, where being re-arrested during the pre-trial period is often used to measure a defendant’s likelihood of recidivism, members of certain groups who end up being arrested at a higher rate will be measured as more likely to recidivate even if differences in arrest rates are not a reflection of true rates of commission but are the result of greater police presence because in the eyes of law enforcement some neighborhoods are viewed as being more dangerous. It is important to note here that these assumptions about the “correct” indicators for assessing a person’s risk of criminal activity rely on considerations of fairness that represent one view of fairness of political institutions among several, which itself is political, contestable, and subject to change (Selbst et al., 2019). Even if the developer is using training data that is representative and accurate and its algorithm is fair according to a statistical notion of fairness, from a normative perspective, we may still have a fairness problem precisely because of these assumptions about risk and how they came to be influential (Mitchell et al., 2021). Ensuring that an algorithm that predicts recidivism is fair under a statistical measure of fairness is a step in the right direction but it is not enough to generate a fair outcome if the criminal justice system construes the risk of recidivism on the basis of biased assumptions and those who are affected by these assumptions are marginalized or excluded from challenging them. What is also needed is a more inclusive system of participation where those who have been marginalized are able to challenge objectionable social structures and societal biases.

This suggests that achieving a more just society overall—especially as this relates to opportunities on the part of historically marginalized to challenge objectionable institutional structures and societal biases—will have an impact on how fairness of predictive algorithms is ultimately assessed. Indeed, a number of influential theories of justice contend that justice is advanced by empowering people to participate on a more equal footing.^22^ If the problem of algorithmic fairness is indeed ameliorated in a society that is more just (according to some appropriate conception of justice), the role of the American public becomes important again. Empowering the historically marginalized to have a greater impact on the institutions and processes that affect their rights and wellbeing is something that must be achieved through collective action on the part of the general polity. When it comes to making algorithms more fair, we all have a role to play.

## 5 Conclusion

Both Congress and numerous states have been active in introducing legislation meant to protect the public from algorithmic discrimination. Unfortunately, these proposed laws have not done a good job in taking a clear position on fairness. Absent such a position, any identifiable group difference can be interpreted as adverse impact, making adverse impact an unavoidable consequence of algorithmic predictions. This can potentially ensnare most if not all predictive algorithms as complicit in unlawful discrimination toward some protected group and underwrite a regulatory regime that is excessively harsh and prohibitive. It is probably safe to say that this is not something intended by the drafters of these laws. A way out of this problem is for these proposed laws to take a clear and explicit position on how they define algorithmic fairness.

Of course, identifying a suitable definition (or set of definitions) of fairness is not a simple task. Even among experts, there’s no consensus as to what counts as a fair result when algorithms are involved. That should not be too surprising: from algorithms to government policies, we invoke fairness as both a moral and a political concept in situations where rights and freedoms are at stake. We all have opinions about what’s fair, and often disagree. But regardless of the challenges, definitions must be chosen and justifiable on reasonable grounds if effective algorithmic fairness regulations are to be established. The objective of such a regime would be to establish a system of laws that can be clearly, consistently, and predictably applied. The challenge to lawmakers is that whatever the ultimate selection turns out to be there will be certain unavoidable complications for the intended regulatory regime. For instance, some definitions may be incommensurable. It already appears that laws regulating AI are going to resemble a mosaic of state and sector-specific laws and regulations. If this regulatory patchwork includes incommensurable definitions, total compliance will be impossible, and we’ll end up with a regulatory system that is incoherent and ultimately doomed to failure. Lawmakers must be cognizant of this possible outcome and take measures to prevent it.

Furthermore, when algorithms are determined to be unfair, lawmakers must understand the problem of defining accountability. Where the unfairness of an algorithm as determined by the selected definition happens to supervene on social facts that have been shaped by historic injustices, making the developer solely legally responsible for remediating and rectifying the resulting unfairness may be hard to justify. More important to this calculus is that placing the burden exclusively on the developer absolves us of our collective responsibility toward achieving a more just society. When we have dealt with similar issues of historic injustice outside the context of artificial intelligence, we have often taken a more diffuse approach to allocating responsibility. While I am not advocating for any particular definition or standard of algorithmic fairness, I am contending that if laws incorporate a definition of fairness where the unfairness is determined by exogenous factors—for instance, where group differences are products of racial injustice—obligations addressing the underlying injustice should follow the model of progressive taxation where responsibility is dispersed across a much broader range of responsible parties. This does not necessarily mean absolving developers of any obligation. The law may still impose specific obligations on developers given the direct role they play. I am simply suggesting that when certain social conditions are implicated in the unfairness of an algorithm, some form of shared responsibility and collective action is the fairer and ultimately the more optimal option. This also has the further advantage of effectuating a more complete approach to algorithmic fairness. Through collective action on the part of the American polity, we can increase opportunities for political participation on the part of those who have been historically marginalized, enhance the heterogeneity of views related to fairness, and gain a deeper understanding of the social context within which predictive algorithms operate. More widespread participation has a better chance of identifying and rooting out factors that stand in the way of achieving a fairer outcome.

Working through the problems outlined here will clarify the issues that lawmakers must contend with when proposing laws meant to regulate algorithms for fairness. Clear answers to these problems will ultimately make for better laws and a more effective regulatory system overall.

## Data Availability

The original contributions presented in the study are included in the article/supplementary material, further inquiries can be directed to the corresponding author.

## Footnotes

^1^The proposed laws cover automated systems. By far the most important of these automated systems are supervised learning predictive models. When discussing AI systems, it is these particular systems and their algorithms that we have in mind.

^2^For federal legislation (see No Robot Bosses Act, 2024; Stop Spying Bosses Act, 2024; AI Consent Act, 2024. For state bills, see New York Artificial Intelligence Bill of Rights, 2023; Boundaries On Technology Act, 2024; Automated Decision Systems, 2024; Safe and Secure Innovation for Frontier Artificial Intelligence Models Act, 2024; Artificial Intelligence Technology, 2024). Other states such as Connecticut, Illinois, Maryland, Massachusetts, New Jersey, Virginia, Vermont, and Washington have proposed similar bills on artificial intelligence.

^3^For a helpful overview, see Mehrabi et al., 2022.

^4^The recent development of President Trump’s revocation of former President Biden’s 2023 sweeping executive order on AI, which directed several federal agencies to develop guidelines on artificial intelligence, may signal a much more hands-off approach to regulation. Although as of the time of this writing we have yet to see what concrete steps will be taken by President Trump regarding proposed federal laws, it is likely that bills that aim at regulating for algorithmic fairness will take a back seat, at least for now. Of course, this still leaves states like California and New York to continue their active role in enacting state-specific AI regulations, which they will undoubtedly do.

^5^See New York Artificial Intelligence Bill of Rights, 2023; Boundaries On Technology Act, 2024; Automated Decision Systems, 2024; Safe and Secure Innovation for Frontier Artificial Intelligence Models Act, 2024; Promoting Ethical Artificial Intelligence By Protecting Against Algorithmic Discrimination, 2024; Automated Employment Decision Tools – Prohibition, 2024; Algorithmic Impact Assessments, 2024.

^6^Take for instance No Robot Bosses Act (2024). Its purpose is to regulate how employers use an automated decision system “derived from machine learning, statistics, or other data processing or artificial intelligence techniques” for making employment-related decisions (No Robot Bosses Act, 2024). The bill prohibits the use of an automated decision system in making an employment-related decision unless the system has had pre-deployment testing and has been verified as meeting a number of conditions including being compliant with applicable employment discrimination laws and not contributing to or resulting in adverse impact. The bill equates adverse impact with group-based discrimination but fails to say what standard of fairness is being violated. States have also been active in introducing legislation aimed at regulating AI. Introduced in the New York State Assembly New York Artificial Intelligence Bill of Rights (2023) aims at safeguarding the rights and protections of New York residents affected by “non-human decision-making systems.” Under this bill, New York residents would be entitled to several key rights and protections, including the right to safe, effective, non-discriminatory systems. The bill requires independent evaluations of automated systems before they are deployed as well as their ongoing monitoring. When it comes to unfair treatment, the bill describes it as “circumstances where an automated system contributes to an unjustified different treatment or impact which disfavors people” on the basis of classifications protected by law (New York Artificial Intelligence Bill of Rights, 2023). There is no explanation of what makes something unjustified. Not to be outdone by the initiatives of other states, the California Assembly introduced A.B. 2,930. Once again, testing is required in the form of impact assessments of automated decision systems to identify reasonable risk of algorithmic discrimination. And as for defining fair treatment, the proposed statute simply proscribes differential impact without providing any explanation as to how the “difference” in differential impact is supposed to be measured (Automated Decision Systems, 2024).

^7^Adverse impact also known as differential impact refers to a disproportionately large negative effect affecting persons on the basis of their protected class membership such as race, color, religion, sex, and national origin.

^8^See Title VII of the Civil Rights Act, 1964; Equal Credit Opportunity Act, 1974; Fair Housing Act, 1968; Americans with Disabilities Act, 1990.

^9^Another way of stating this point is to say that statistical parity on its own is not good enough to define fairness because it fails to account for situations where differences in prediction rates are perfectly justified, which do exist as our intuitions about fairness suggest.

^10^See Promoting Ethical Artificial Intelligence By Protecting Against Algorithmic Discrimination, 2024; New York Artificial Intelligence Bill of Rights, 2023.

^11^COMPAS risk scores ranging from 5 to 10 indicate a higher risk of recidivism and therefore an operationally salient factor when deciding whether or not to deny a defendant parole. Scores below the 5-point threshold signal low risk and defendants assigned scores below it are essentially treated as non-recidivists.

^12^Equalized odds also goes by other names. See Berk et al. (2017) who use the term “conditional use-accuracy equality.”

^13^Predictive values are a statistical measure used to assess the accuracy of an algorithm’s predictions. Where an algorithm’s classification is binary, each instance is assigned to either a positive or negative class. The positive class is typically the condition being tested for—in COMPAS’s case, the instances of its risk scores classifying individuals as recidivists. The negative class is all of the instances where COMPAS predicts individuals as non-recidivists. By distinguishing positive predictions from negative ones, we can direct our attention to each class separately and evaluate their respective accuracy.

^14^For a discussion of types of fairness definitions (see Verma and Rubin, 2018; Barocas et al., 2023; Corbett-Davies et al., 2017; Dwork et al., 2011; Joseph et al., 2017; Kearns et al., 2018; Zafar et al., 2017).

^15^A further assumption behind the impossibility of simultaneously equalizing positive predictive values and error rates is that the algorithm is not 100% accurate in its predictions. COMPAS surely fits that bill – it is nowhere close to being perfectly accurate. And when it comes to predicting complex human behavior, no algorithm is capable of achieving such accuracy.

^16^For an important discussion of this issue (see Chouldechova, 2016; Kleinberg et al., 2016; Hardt et al., 2016).

^17^One approach to dealing with the problem of incommensurability is to suggest that developers should not be expected to perfectly satisfy the requirements of such definitions. Instead, they should satisfy them approximately. In other words, we are envisioning a kind of a trade-off between these definitions. The difficulty with this is that even if we place the trade-off on the Pareto frontier, there will be a range of options to choose from. The question then becomes, which trade-off profile do we select on the Pareto frontier? This itself requires that we assess the selection from the standpoint of fairness. Which profile would be the fairest? This is not an easy question to answer and may require a more involved process for selecting the appropriate profile, which may differ from one case to another. Such an approach may tip things in the direction of government oversight where federal or state agencies are involved in the selection process. That of course will entail a more complex regulatory system, one akin to the FDA’s approval process over medical equipment or drugs, for instance. The proposed laws as drafted do not envision such a complex system.

^18^Along these lines, it may also be argued that some of this responsibility should be shared by those further down the supply chain who supply or deploy the algorithm. But the primary agent of responsibility remains the developer.

^19^Datasets used in the development process often come with their own problems. They may not be representative of certain groups, infected by implicit biases, etc. Moreover, the developer is often not responsible for creating these datasets. Nevertheless, we still think the developer has agency over the data. The developer can check the quality of the data, take remedial steps to correct problems such as underrepresentation, or if the datasets are hopelessly compromised, can choose not to use them.

^20^These assumptions characterize a typical situation for a couple of reasons. First, a rational developer is interested in enhancing the commercial appeal and value of its algorithm, and maximizing the algorithm’s accuracy (in terms of its predictive values) across all groups and to the same extent advances this goal. In other words, the rational developer strives to develop an algorithm that’s well-calibrated. But that means the algorithm the rational developer strives to develop, where there are differences in base rates, will tend toward equality of predictive value and away from equality of error rates. What’s more, well-calibration often results from supervised learning, which is a common approach in machine learning used by developers to build predictive algorithms.

^21^Although I am agnostic regarding which definitions of fairness should be codified in law, there is some advantage to selecting fairness definitions for which accountability may be straightforwardly attributed to the developer––e.g., parity of positive predictive value. The benefit of choosing parity of positive predictive value as the operative definition is that racial disparities underlying differences in prevalence rates may still be simultaneously addressed, except in this case the responsibility would fall on the shoulders of general society. Making inroads in narrowing racial differences in recidivism rates through broad-based participation has the virtue of diminishing differences in error rates. And so by instituting a division of labor where both developers and the general public have a constructive role to play, we may be able to effectuate a regulatory regime that is capable of tackling a broader range of algorithmic unfairness.

^22^Nussbaum (2011) for instance argues that having capabilities that enable one to have political agency is essential to human dignity, for they ensure that people have a voice in shaping their society. (Dworkin, 2000) on the other hand argues that individuals must have equal access to resources that enables them to exercise their fundamental rights and freedoms. Still others like (Arneson, 1989) contend that equality of opportunity is the bedrock of a just society.
